# Variation of Adolescent Snack Food Choices and Preferences along a Continuum of Processing Levels: The Case of Apples

**DOI:** 10.3390/foods8020050

**Published:** 2019-02-01

**Authors:** Elizabeth Svisco, Carmen Byker Shanks, Selena Ahmed, Katie Bark

**Affiliations:** 1 Food and Health Lab, Montana State University, Bozeman, MT 59718, USA; sviscoe@gmail.com (E.S.); selena.ahmed@montana.edu (S.A.); 2 Montana Team Nutrition; Montana Team Nutrition, Montana State University, Bozeman, MT 59718, USA; kbark@mt.gov

**Keywords:** nutrition, processed, snack food, food, ingredients, preferences, eating behaviors, healthy snacks, adolescent snacking

## Abstract

Food processing is used for transforming whole food ingredients into food commodities or edible products. The level of food processing occurs along a continuum from unprocessed to minimally processed, processed, and ultra-processed. Unprocessed foods use little to no processing and have zero additives. Minimally processed foods use finite processing techniques, including drying, freezing, etc., to make whole food ingredients more edible. Processed foods combine culinary ingredients with whole foods using processing and preservation techniques. Ultra-processed foods are manufactured using limited whole food ingredients and a large number of additives. Ultra-processed snack foods are increasing in food environments globally with detrimental implications for human health. This research characterizes the choices, consumption, and taste preferences of adolescents who were offered apple snack food items that varied along a processing level continuum (unprocessed, minimally processed, processed, and ultra-processed). A cross-sectional study was implemented in four elementary school classrooms utilizing a buffet of apple snack food items from the aforementioned four food processing categories. A survey was administered to measure students’ taste acceptance of the snacks. The study found that the students selected significantly (*p* < 0.0001) greater quantities of ultra-processed snack foods (M = 2.20 servings, SD = 1.23) compared to minimally processed (M = 0.56 servings, SD = 0.43) and unprocessed (M = 0.70 servings, SD = 0.37) snack foods. The students enjoyed the taste of ultra-processed snack foods (M = 2.72, SD = 0.66) significantly more (*p* < 0.0001) than minimally processed (M = 1.92, SD = 1.0) and unprocessed (M = 2.32, SD = 0.9) snack foods. A linear relationship was found between the selection and consumption quantities for each snack food item (R2 = 0.88). In conclusion, it was found that as processing levels increase in apple snack foods, they become more appealing and more heavily consumed by elementary school students. If applied broadly to snack foods, this conclusion presents one possible explanation regarding the high level of diet-related diseases and nutrient deficiencies across adolescents in America. Food and nutrition education, food product development, and marketing efforts are called upon to improve adolescent food choices and make less-processed snack food options more appealing and accessible to diverse consumers.

## 1. Introduction

Processed foods have increasingly dominated the United States’ food supply, food environments, and diets over the past five decades [1] with detrimental implications for nutrition and human health [2,3,4,5]. The trend of increased processed food consumption characterizes the nutrition transition as a shift towards nutrient-poor diets filled with ultra-processed food items [2]. The overconsumption of ultra-processed foods that are often formulated using saturated or trans fats, excess sugar, excess salt, and artificial ingredients has had negative implications for diet-related health outcomes, including increased incidence of obesity, Type 2 Diabetes, cardiovascular disease, and cancer [2,3,4,5]. One in five children ages 6 to 19 and living in the United States are classified as obese with a body mass index at or above the ninety-fifth percentile [6]. A recent article demonstrates that a 10% increase in consumption of ultra-processed foods among study participants was associated with a significant increase (greater than 10%) in overall cancer and breast cancer [5]. Along with the United States, the nutrition transition and associated nutrition and health outcomes are occurring in communities globally [7].

Ultra-processed foods comprise 58% of the consumed calories and 90% of the added sugar intake in the American diet [1]. It was found that meals prepared with ultra-processed and processed food ingredients had one third more added sugar, one fourth more saturated fat and sodium, less than half the fiber content, and two thirds more calories when compared to meals prepared from the processed and minimally processed groups [4]. For adolescents, regardless of country of residence, obesity was positively correlated with the taste of fat and sugar-enriched food products [8]. Eliminating ultra-processed products from a diet can improve consumer health with studies reporting decreases in attention deficit hyperactivity disorder (ADHD) symptoms [9], a reduction in insulin resistance in children with Type 2 Diabetes [10], and decreased mineral deficiencies in children with autism [11].

Food processing (Table 1) can be characterized at different levels along a continuum from unprocessed, minimally processed, and processed culinary ingredients up to processed foods and ultra-processed foods. The different levels of food processing vary according to the degree of manipulation including the utilization of new technologies and the input of artificial ingredients that take foods further from their natural state [1]. At the extreme end, ultra-processed food items often have little physical resemblance to the fresh and wholesome food items that they were originally derived from [12]. The addition of extra processing steps and artificial ingredients transforms food products such as meats, fruits, and vegetables into new foods that can be both visually and nutritionally different compared to their original whole food state [4]. Unprocessed foods include fresh fruits, vegetables, and meats that have undergone limited processing including cleaning, portioning, chilling, grating, etc. [13]. Minimally processed foods have undergone a small amount of processing such as drying, freezing, or fermentation [13]. Processed culinary ingredients include sugar, oil, fat, salt, and other ingredients that are originally taken from plants and then milled, pressed, or pulverized to be used in cooking or baking [13]. Processed products are whole food ingredients that have been combined with processed culinary ingredients using processing methods along with preservation techniques such as salting, pickling, smoking, curing, etc. [13]. Lastly, ultra-processed foods are created using ingredients that are not used in culinary work [1], including artificial flavors, colors, sweeteners, emulsifiers, and preservatives. Ultra-processed products can be fortified with micronutrients and are formulated to be “ready-to-eat” versions of whole food items [13].

Given that snack foods are a large part of the adolescent diet and impact health outcomes, it is important to understand adolescent preferences for snack foods along a continuum of processing. Currently, there is limited research that investigates adolescent preferences for snack food items from various processing levels. This study seeks to address the aforementioned research gap by examining adolescent food choices, consumption, and taste preferences for snack food items from the continuum of processing from unprocessed to minimally processed, processed, and ultra-processed. The overall research question of this study is: *What are the food choices, consumption, and taste preferences of adolescent students who are offered snack food items that vary along a continuum of processing levels (unprocessed, minimally processed, processed, and ultra-processed)?*

It is hypothesized that the study participants will select and consume greater quantities of the processed and ultra-processed apple snack food items when compared to the unprocessed and minimally processed snack food items on the basis of their food preferences. The findings from this study have the potential to increase the awareness of the impact of food processing on the incidence of obesity and chronic illness and decrease the consumption of ultra-processed foods in America. Through a clear understanding of what ultra-processed snack foods are, how they impact consumption and taste preferences, and potential health implications from overconsumption, consumers will hopefully be encouraged to choose less-processed foods for their diet. The findings from this study also have the potential to enhance and support health, nutrition education, and school foodservice programs by providing them with concrete results regarding ultra-processed snack food preferences and consumption.

### Background

Processed and ultra-processed foods are often high in calories while being low in nutritional value [14]. Ultra-processed foods are void of naturally occurring ingredients and are not fresh food items [4]. Ultra-processed foods are characterized as rich in solid fats, sugar, sodium, artificial ingredients, additives, and chemicals instead of protein, fiber, vitamins, and minerals [15,16]. The ingredients in ultra-processed food products are added for enhanced taste, visual appeal, or preservation [14]. Examples of ultra-processed ingredients include high fructose corn syrup, trans fats, monosodium glutamate, artificial colors, brominated vegetable oil, artificial sweeteners, and nitrates/nitrites.

The top five food companies in the United States responsible for the sales of packaged foods (Kraft, PepsiCo, Nestle, Mars, and Kellogg) control approximately 25% of US food sales [15]. These food companies are a part of the processed food industry that manufacture ultra-processed foods including lunch meats, sweetened beverages, candy bars, cereals, and snack bars. For example, Kraft Foods is responsible for 6.8% of all packaged food sales in the United States [15]. The prevalence of processed and ultra-processed foods in diets in the United States has rapidly increased due to key food environment characteristics including enhanced desirability (such as flavor and appeal), affordability, convenience (such as a decreased preparation time, portability, and an extended shelf-life), and efficiency [17,18].

It is well recognized that food processing has the potential to enhance the sensory desirability of foods by altering their physical structure, taste, aroma, and texture [17]. With the development of new food processing technologies coupled with consumer demand, the number of processed products available has notably increased as chemists continue to develop and add flavor enhancers and other artificial ingredients to foods [19,20]. For example, in the 1960s, processed potatoes, including potato chips, made up 35% of potato use and today this number has increased to 64% [21]. Research supports that ultra-processed foods have addictive qualities, which stems from hyper-palatable taste and unnaturally high levels of pleasure associated with consumption that ultimately impact behavioral characteristics and can lead to overeating [22]. The addictive qualities of ultra-processed foods are magnified at a young age and are heavily influenced by an early introduction to them along with a high frequency of consumption [22]. Child-focused marketing has increased this issue. For example, in 2012 4.6 billion dollars was spent on fast food advertising and 6–11-year-old children viewed an average of 3.2 fast food advertisements per day [23].

The most frequently consumed ultra-processed foods by Americans include packaged snack foods such as cookies, salty snacks, and breakfast cereals [12]. Research demonstrates that snacking is on the rise and predicts future increases in the consumption of ultra-processed snack food items [24]. Specifically, between 2016 and 2017, the number of Americans snacking five or more times per day increased from 11.5% to 14.2% [24].

Today, adolescents have greater exposure to highly processed snack foods than previous generations. A recent study demonstrated that adolescents consume an average of 4.3 processed snacks per day [25]. From 1977 to 1978, adolescents consumed about 300 calories per day from snacks; this number increased to 526 calories from 2005 to 2006 [26]. These 526 calories from snacks make up between 22 and 38% of the recommended daily calories for an adolescent female and between 16 and 33% for an adolescent male, depending on their activity level and exact age [27]. The key drivers of adolescent food choices for snacks include convenience, accessibility, and desirability (i.e., pleasurable to consume during social or leisurely times) [28].

The production of ultra-processed foods presents challenges to the sustainability of food systems and sustainable diets because of the energy and materials required for their processing and packaging. Overall, the production of food requires 10% of the total U.S. energy budget, 80% of freshwater, and 50% of all U.S. land [29]. At the same time, food production represents food waste challenges due to food loss on farms, in retail stores, in food operations, and in households as a result of inefficiency throughout all stages in the food system [29]. In 2008, it was found that food processing specifically led to 16% of food loss during manufacturing [29]. Snack food packaging is designed to preserve, contain, trace, and increase the appeal of snack food items for Americans. Packaging innovations are leading to heightened quantities of waste ending up in landfills [30]. In 2010, 21% of the total waste generation was made up of food while 16% was from paper, 5% was from glass, and 17% was from plastics, which are all used in food packaging [31]. This solid waste leads to chemical leaching into the soils (hence poisoning soils for food to be grown in) or groundwater and increased greenhouse gases being released into the atmosphere [30].

The increased consumption of ultra-processed foods and associated health outcomes is driving a need for more nutrition education regarding ultra-processed foods as well as the development and marketing of less-processed snack foods. The five stages of Popkins’ [2] nutrition transition highlight the need for a shift from dietary patterns characterized by processed foods and high sugar and fat consumption to desired dietary patterns that include the reduced consumption of processed foods coupled with increased consumption of fruits and vegetables. This change in dietary patterns away from high fat and sugar processed foods is associated with reduced body weight along with a reduced risk for diet-related chronic disease (obesity, Type 2 Diabetes, cardiovascular disease, and cancer) that ultimately extends aging and improves overall health [2,3,4,5]. In order to foster the desired societal and behavior change away from the consumption of ultra-processed foods, nutrition education efforts are increasing regarding the impacts of ultra-processed foods and benefits of healthy snack foods, particularly fruits and vegetables [31]. The United States Department of Agriculture and MyPlate promote sliced vegetables, fresh fruits, dried fruits, and unsweetened applesauce as healthy snacks for children along with lean proteins, nuts, and other whole food items [32]. Fruit and vegetable consumption provides people with dietary fiber, which can lower obesity rates and the incidence of cardiovascular disease [31]. Vitamins, minerals, and phytochemicals, essential for human health, are also supplied through fruits and vegetables and can produce anti-inflammatory, antioxidant, and phytoestrogen properties to support bodily functions [31]. Companies are learning and responding to consumer demand and nutrition research by developing and marketing less-processed food snacks that better support desired nutrition and health outcomes. Examples include fruit and nut trail mix with zero added sweeteners or preservatives, sweet potato tortilla chips made from only three whole ingredients, fruit snacks with only fruit as an ingredient, and dried chickpeas [24].

## 2. Materials and Methods

The cross-sectional research examined adolescent food choices for unprocessed, minimally processed, processed, and ultra-processed versions of apple snack food items. Adolescent students were presented with a one-time, unlimited buffet of four apple snack food options. Each buffet included four of eight apple snack foods (two varieties of raw apples, two brands of dried apple slices, two brands of cinnamon applesauce, and two apple-based fruit snack brands) to reduce bias within and between the processing types. Adolescent snack food choices were measured by student selection, consumption, and taste preferences. The selection and consumption data were measured through food weight while food preference data was measured through a survey.

### 2.1. Subjects

The data were collected at three schools in Southwest Montana and included participation from four fourth grade classrooms made up of 25, 28, 24, and 20 students, respectively, for a total of 97 research subjects. The data were collected during class periods lasting 45 minutes each. Middle schools were selected based upon a convenience sample of middle schools within a one-hour driving distance for the research team. The middle school principals and teachers in Southwest Montana were contacted by the research team with the study details. Several schools declined to participate because research initiatives did not align with the schools’ individual research initiatives. The willing principals and teachers were asked about the number of nine to ten-year-old students within the middle school and the number of fourth grade classrooms that could potentially participate in the study. The ideal class size was approximately 25 students per class. Three middle schools were selected and they consented to participate. The participating ages and grade level were selected to fill a gap in sensory research that targets participants in the beginning stages of adolescence.

Consent was given by the principal of each school and each individual student. The principals were able to review the methodology and content of the study. According to the school’s protocol for research, verbal consent was given by each student after a document was read aloud to the participating class. The consenting information outlined the study purpose and methods and that the participation was voluntary. Students were excluded if they were not the correct age, were allergic to any of the food products, or were not willing to participate.

The table below outlines the descriptive characteristics of the schools where students involved in the study were enrolled (Table 2). The demographics between schools varied, with School 1 having 217 total students with 92.7% Caucasian, 51% males, 49% females, and 17.51% of students participating in the National School Lunch Program [33,34]. Two fourth grade classes were used at School 1. School 2 included 199 students with 87.6% Caucasian, 58% females, 42% males, and 10.6% of students participating in the National School Lunch Program [33,34]. School 3 included 448 students with 89.1% Caucasian, 52% males, 48% females, and 37.1% participating in the National School Lunch Program [33,34].

Adolescence is defined from 10 to 19 years of age [35]. The test subjects were between nine and ten years old. This age group encompasses the beginning stages of adolescence, an important age group to target when examining the formation of snack food choices [36]. It is critical for adolescents to establish healthy snacking habits going into early adulthood.

### 2.2. Snack Food Product Samples

The research study included eight different apple-based snack food items with two types selected for each processing level (Table 3). Apples were selected as the main food item because they are within the top two most-consumed fruits annually for youth in America [37]. Specifically, out of the top ten consumed fruits, raw apples are the second most consumed and applesauce is the seventh most consumed.

Red Delicious and Gala apples were selected because they are the top two most-produced apple varieties in America, with more than 54 million 42-pound units of Red Delicious apples produced in 2011 and nearly 33 million 42-pound units of Gala apples produced in 2011 [40]. Bare Dried Apple Chips and UNFI Dried Fruit Apples were selected as minimally processed snack food items because Bare Apple Chips are widely accessible to consumers while being sold at some of the nation’s largest retail stores including Walmart [41]. UNFI Dried Apples are sold by a large distributing company called United Natural Foods that distributes to grocery stores across the United States [42]. Musselman and Seneca brand cinnamon apple sauces were chosen because they are widely distributed throughout America as snack food items and can be purchased at some of the nation’s largest retail chains including Walmart [41]. Western Family Fruit Snax and Welch’s Apple Medley Fruit Snacks were chosen because Welch’s is among the top five fruit snack brands in America [43] and both brands are well-known in the United States while having numerous channels to connect their snack foods to many Americans.

Only one brand item from each processing group was available to the students in each classroom for the research study, which equaled four products being tested at once. The eight apple snack food items and their ordering were randomized so each classroom received a different buffet spread.

The grocery stores from which the food items were purchased were randomly selected from a list of local food outlets that sold the desired product. At the selected grocery stores, whole apples were selected to represent similar shape, size, and coloring, forming a uniform sample for the research subjects. At the selected grocery stores, apple chips, applesauce, and fruit snacks were selected with the furthest expiration dates so that the products were fresh for the study participants.

### 2.3. Basic Protocol

The data collection times were scheduled apart from meals or snacks so that hunger levels did not influence the amount of food that was taken or consumed by study participants. The consenting process included a signed letter of approval from all school principals involved that stated parents were informed of the research study, approval of an expedited Institutional Review Board from Montana State University (approval ES102517), and a verbal message relayed to student participants. This message was read to all classrooms before the study began and informed students that their information would be anonymous and that they did not have to participate in the study if they did not wish to. The teachers, principals, and students were informed of what foods were being sampled in order to avoid issues with food allergies and protect the students. To further ensure the safety of the students, all raw items were washed, disposable gloves were worn by team members handling food, surfaces were wiped down, and serving utensils were available to avoid contamination of food items. All four snack food items were placed in bowls and put on the buffet line in front of the classroom. Apples were sliced instead of whole due to the greater appeal for sliced fruit versus whole fruit when working with children [44]. The whole apples were sliced using an apple slicer to create 6 equal-sized wedges per apple, which increased uniformity and efficiency in preparation. The dried apples, applesauce, and fruit snacks were placed in similar bowls. Fruit snack packages were individually opened and poured in a bowl so that packaging did not influence the decisions. The bowls were filled with enough snack food item for two serving sizes for each student, as specified by the Nutrition Facts panel. More was added if a classroom ran out and extra weight of new product was accounted for. The snack food labels were used to determine the serving sizes for each product; one serving size was equivalent to one fruit snack bag (27 grams for Western Family and 24 grams for Welch’s), 1/2 cup of dried apple chips (18 grams for Bare Brand and 30 grams for UNFI), 1/2 cup of applesauce (118 grams), and 1/2 medium apple per student (4 slices or 84 grams).

Four snack food bowls were placed on scales to weigh portion sizes selected by the students. Signs were placed in front of each bowl to label them with either a “plain” or “catchy” name in order to follow the behavioral economics techniques of “using catchy names” and “using signage” to increase the selection and consumption of food items [45]. Both the signage types were randomly assigned to 2 classrooms each. The snack food “catchy names” were created by a fourth-grade student. All the names are shown below in Table 4.

**Figure 1:**
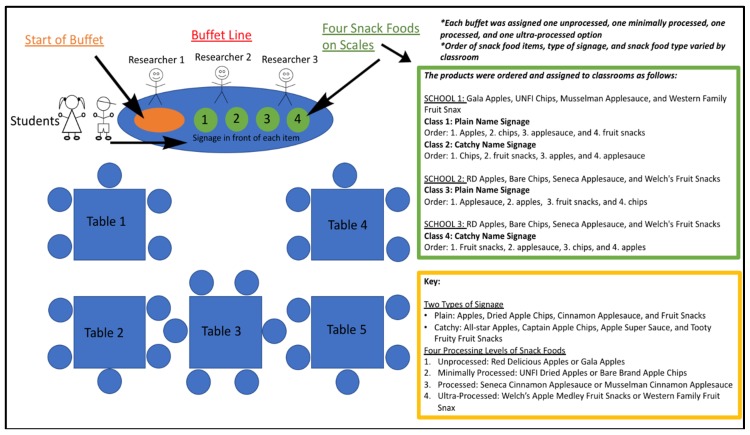
The figure above displays the classroom setup of the research and explains how snack food items were assigned, ordered, and presented to the students based on the classroom number.

The four snack foods were compared with each classroom of students to capture all the levels of food processing and compare preferences when all the options were available to the research subjects [46,47,48]. Each student was assigned a number, which was written on their plate and bowl and then used to record and match the student selection, consumption, and survey results. The researchers handed materials to students and recorded the weight in grams on the scale for each snack food item as each student selected their portion. The students were told not to share their food and that they did not have to finish everything on their plates and in their bowls. Figure 1 summarizes the overall design and outline of the research setup.

After the students finished snacking, they were asked to bring their plates and bowls (with leftover food) to the weighing station where the researchers measured the waste in grams for each product individually. To weigh the applesauce, an empty bowl was tared on the scale and then each student’s bowl was placed on the scale with the remaining applesauce in it to get the accurate weight of the applesauce alone. To weigh the three snack food items on the plate, a plate was tared on the scale and then each item from each student’s plate was scraped onto the plate individually to weigh it.

Next, the subjects were asked to record their plate number on and complete a survey to rate their satisfaction for all four food products (Table 5). The “Tried It, Liked It, Loved It” survey was based upon a survey initially developed by Montana Food Corps [49] and then modified by researchers at the Montana State University Food and Health Lab and Montana Team Nutrition and validated in various school nutrition studies [50].

### 2.4. Data Analysis

All data was entered into Excel (Microsoft Excel, Redmond, WA, United States of America, 2017). This included the survey results, which were coded using numbers to represent “did not try,” “tried,” “liked,” or “loved” in order to analyze the results. Data were also categorized in Excel based on the use of either “plain” or “catchy” names. A one-way ANOVA test (analysis of variance used to analyze differences among group means in a sample) was used to compare taste preferences to the selection and consumption data (percentages of a serving) and to the two labeling types (plain or catchy names) after confirmation of normal distribution was complete. All calculations in the table below (Table 6) were made and recorded in an Excel spreadsheet.

Data from schools were aggregated. JMP (JMP^®^ SAS Institute Inc., Cary, IL, USA) was used to look for descriptive statistics and correlation within the results. Descriptive statistics were used to calculate the means, ranges, and standard deviations for the selection data, consumption data, and survey responses. A one-way ANOVA test was used to compare taste preferences to the selection and consumption data (percentages of a serving) and to the two labeling types (plain or catchy names) used. Significance was set at *p* ≤ 0.05.

## 3. Results

### 3.1. Snack Food Selection

Students selected significantly more fruit snacks (ultra-processed food) compared to the other three snack food options (processed, minimally processed, and unprocessed) based upon comparing the percentage of a serving that was selected by each student for all snack food items (*p* ≤ 0.0001). A mean of 70.5% of a suggested serving size was selected for the unprocessed snack food (standard deviation of 0.37), 56.3% of a suggested serving size was selected for the minimally processed snack food (standard deviation of 0.43), 87.6% of a suggested serving size was selected for the processed snack food (standard deviation of 0.49), and 202% of a suggested serving was selected for the ultra-processed snack food (standard deviation of 1.23). This means that the subjects selected the greatest quantities of ultra-processed fruit snacks and the smallest quantities of minimally processed dried apple chips. The signage type of “plain” versus “catchy” names did not create a significant difference in food selection (*p* = 0.9590).

### 3.2. Snack Food Consumption

**Figure 2:**
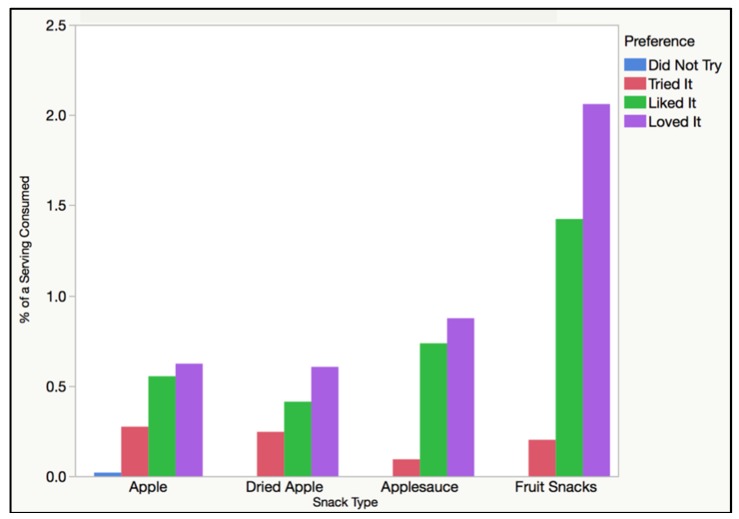
The percent of a serving consumed is detailed based on the four food processing levels and snack food options available to the students. It is important to note that since the selection had a linear relationship with consumption, the figure comparing selection to snack type was nearly the same as the one for consumption.

The students consumed significantly more fruit snacks (ultra-processed food) compared to the other three snack food options (processed, minimally processed, and unprocessed) based upon comparing the percentage of a serving that was consumed by each student for all snack food items (*p* ≤ 0.0001). A mean of 53% of a suggested serving size was consumed for the unprocessed snack food (standard deviation of 0.38), 40% of a suggested serving size was consumed for the minimally processed snack food (standard deviation of 0.36), 72% of a suggested serving size was consumed for the processed snack food (standard deviation of 0.52), and 186.7% of a suggested serving was consumed for the ultra-processed snack food (standard deviation of 1.15). Therefore, the students consumed the greatest quantities of ultra-processed fruits snacks and the smallest quantities of minimally processed dried apples. Note that the students selected and consumed less minimally processed snack foods (dried apple chips) than unprocessed snack foods (apple slices). Besides this comparison between unprocessed and minimally processed snack foods, the selection and consumption increased as the processing continuum increased from unprocessed to ultra-processed. The signage type of “plain” versus “catchy” names did not create a significant difference in food consumption (*p* = 0.7712). Figure 2 below shows how processing levels affected snack food consumption levels.

**Figure 3:**
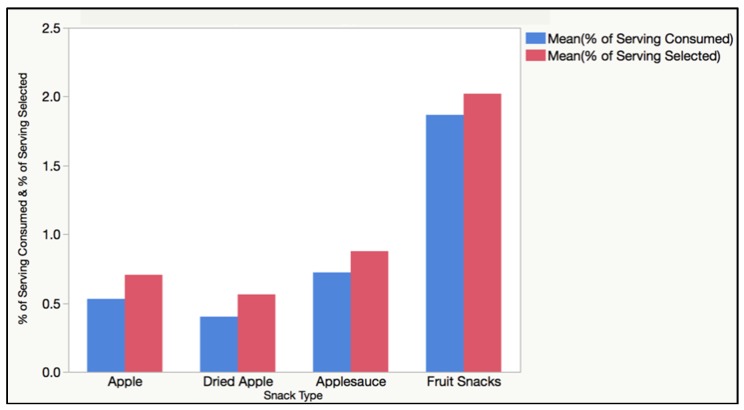
Linear relationship between the apple snack food selection and consumption.

A linear relationship was found between the selection and consumption quantities for each snack food item (R2 = 0.88, *p* < 0.0001). For all four processing categories, the amount of food selected directly influenced the amount of food that was consumed (Figure 3). Table 7 below outlines the selection and consumption data results for each snack food type and also lists the serving sizes used as references when calculating percentages.

### 3.3. Snack Food Preferences

**Figure 4:**
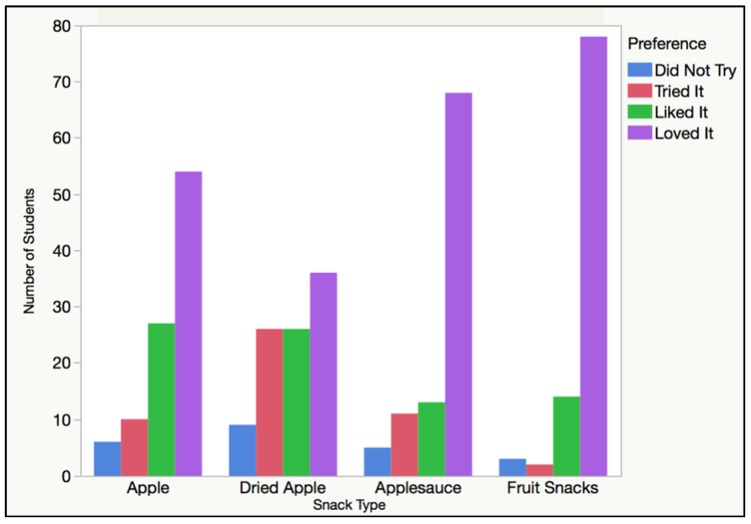
Tried It, Liked It, Loved It Survey results that portray the number of students who did not try, tried, liked, or loved each apple snack food for the four processing levels.

Student preference was calculated using the survey results and a coding scale where 0 = did not try, 1 = tried it, 2 = liked it, and 3 = loved it. Therefore, when all the survey results were combined on JMP for each snack food item or processing level, a mean closer to three exemplified higher subject taste satisfaction while a mean closer to 0 exemplified lower subject taste satisfaction. The students enjoyed the taste of ultra-processed snack foods (mean of 2.72 with a standard deviation of 0.66) significantly more (*p* < 0.0001) than other processing levels including the processed snack foods (mean of 2.48 with a standard deviation of 0.89), minimally processed foods (mean of 1.92 with a standard deviation of 1.0), and unprocessed foods (mean of 2.32 with a standard deviation of 0.9). In accordance with the selection and consumption results, satisfaction increased moving upward on the processing continuum, except when comparing the unprocessed and minimally processed categories (Figure 4). More specifically, for the unprocessed snack food option, 6.2% of the students did not try it, 10.3% tried it, 27.8% liked it, and 55.7% loved it. For the minimally processed snack food option, 9.3% of the students did not try it, 26.8% tried it, 26.8% liked it, and 37.1% loved it. For the processed snack food option, 5.2% of the students did not try it, 11.3% tried it, 13.4% liked it, and 70.1% loved it. For the ultra-processed snack food option, 3.1% of the students did not try it, 2.1% tried it, 14.4% liked it, and 80.4% loved it. The highest percentage was found for subjects who loved fruit snacks (80.4%) and the lowest “loved it” result was found for the dried apple chips (37.1%). These numbers are listed below in Table 8.

## 4. Discussion

Prior to this study, several research gaps existed regarding youth taste preferences which the current study has contributed to addressing. Previous sensory studies often focus on a limited age range with most involving either babies and toddlers or adults. Though there are published studies on sensory analysis conducted with toddlers and preschoolers [51,52,53], fewer sensory studies involve youth that are in the elementary, middle, and high school age range. For example, a study involved children between the ages of five and ten along with their mothers as study participants to compare preferences for creaminess and perception of fat in foods [54]. As flavor preferences may differ depending on exposure to different food items throughout the lifespan or nutritional goals based on growth and development, it is critical to evaluate taste preferences across all ages.

There are many research studies that focus on preferences for fruits and vegetables. For example, a study compared the taste of various vegetables after preparing them using different cooking styles or methods in order to determine how this influenced preference [55]. Another study provided children and adults with a familiar fruit and a novel fruit to compare appetitive and familiarity ratings by sensory stages [56]. The focus on fruit and vegetable research is a result of current US Dietary Guidelines to increase intake [57]. Though there are studies focusing on fruit and vegetable snacks, it was determined that further research needed to be conducted with grade school youth and snack food items to understand adolescent food preferences for these snacking options.

Two research studies were found that compare the taste of ultra-processed food products to less-processed food products. One taste test study involving a processed food product aimed to determine whether children perceived food with nutrition claims on their labels as healthier or tasting differently [58]. This study focused on how marketing and packaging affect taste rather than how ingredients and processing affect taste. The participants were asked which product was healthier and which tasted better when two identical products were placed in front of them (one involving a health claim and one without) [58]. Another study compared the taste and acceptance of whole-grain versus refined pancakes and tortillas to see if whole-grain options could replace refined products for grade school children [59]. The study was voluntary at lunch and compared these options by asking participants about overall liking, taste, color, softness, and ranking using a hedonic facial scale [59]. To examine consumption and connect consumption to preference, plate waste was collected [59]. In this study, no differences were noted in consumption of whole-wheat pancakes when compared to refined wheat pancakes, while consumption of whole-wheat tortillas was lower than refined products [59]. Though the second study did compare an ultra-processed product to a less-processed one, it involved lunch products and did not involve snack food products.

Research has also been conducted to compare various components associated with ultra-processed foods including intensity of sugary or salty flavors or preference for high-fat products. A study using 4- to 6-year-old children failed to confirm that the children who are sensitive to bitter tastes would report a higher intake of sweets and a lower intake of savory fats [60]. This study included four one-hour taste tests at dinnertime along with a final taste test including measurement of body composition [60]. Another study examined children and their mothers’ preferences for creaminess and perception of fat in pudding along with concentrations of sucrose in water [54]. It was found that children preferred higher sucrose amounts in water and lower fat content in pudding compared to their mothers [54]. The methodology of this study included having participants taste different concentrations of sucrose and fat in water and pudding and then rank the samples based on intensity of sweetness and creaminess [54]. Overall, it is evident that more research needed to be conducted involving children throughout adolescent years and taste preferences associated with ultra-processed snack foods versus less-processed snack foods.

The *Variation of Adolescent Snack Food Choices and Preferences along a Continuum of Processing Levels: The Case of Apples* study was structured in such a way that it would address the gaps in research and provide new insight. As with the studies mentioned above, this study incorporates a taste test, plate waste data, hedonic rating for the taste of food items, and a variety of processed snack food products to make comparisons. Like the tortilla study mentioned above, it was found that the participants were drawn to highly processed food items and consumed more of them [59]. It was also found that the participants were drawn to snack food items that were more highly processed, which could have had to do with taste qualities associated with sugar, salt, and fats [54,60]. Therefore, this research builds upon prior research and expands the realm to include the following results.

This research study provides evidence about the snack food preferences of adolescents along a continuum of unprocessed, minimally processed, processed, and ultra-processed snack foods. The key findings from this study highlight the following behaviors and preferences of the study participants regarding snack food consumption: the participants selected and consumed more processed and ultra-processed snack foods when given an array of snacking options, the participants enjoyed the taste of ultra-processed and processed snack foods when compared to less-processed options of similar flavor, and the participants consumed greater quantities of snack foods when they selected greater portion sizes. Strategies are necessary to increase the desirability of less-processed snack food options that have higher nutrient density, fiber, and water content.

This study demonstrates that over two suggested servings of fruit snacks were consumed on average by students while half a serving or less of apples and dried apples were consumed. The correlation of snack food choice with taste preference suggests that the research subjects consumed high amounts of ultra-processed foods in order to please their palettes and hunger levels. The survey results highlight that over 80% of the students loved the taste of ultra-processed fruit snacks while only 55% loved the apple slices and 37% loved the dried apples. Since taste has an influence on food choices [22], these results show that adolescents are drawn towards the flavors and textures of more-processed products. The hyper palatable flavors associated with ultra-processed foods come from salts, sugars, fats, and additional additives. The artificial additives that can enhance flavor include high fructose corn syrup, mono sodium glutamate (MSG), and other chemically-derived ingredients [61,62].

When looking at the selection and noticing that higher quantities of ultra-processed and processed snack foods were chosen by subjects before tasting them, the participants likely associated more-processed products with better taste before trying them. Previous exposure to these snack food items or the physical appearance of the items may have influenced the participants’ food choices. For example, the bright colors, shapes, and gooey texture associated with fruit snacks or the smooth, mashed texture of applesauce may have encouraged students to select more of these items. Also, the noticeable cinnamon flavor (smell, cinnamon speckles, and physical sign) of the applesauce could have swayed students to select less or more of it due to previous exposure to cinnamon and how much they enjoy the spice. Being able to eat the applesauce out of a bowl with a spoon also gives the snack food item a unique appeal. Another component that could have influenced selection is peer pressure. It was noticed that students became particularly excited about fruit snacks and relayed this excitement to their peers. Noticing how much was selected for each snack food product by peers and hearing their comments about the items could have encouraged them to select more or less of particular options.

When physically comparing fresh apple slices to fruit snacks or dried apples, a serving size of fresh apples takes up a much larger visual space due to water and fiber content in whole apples. Additionally, the water and fiber content have the ability to nourish the body and satiate hunger. It was also noted that in every class involved in the study, at least one student asked for a second serving of the fruit snacks after all the students had served themselves at the buffet line. Out of the four snack food options, the fruit snacks were the only snack food item that the students continued to ask for more of. It was also the only snack food item that needed to be refilled on the buffet line by the research team due to high levels of student selection.

Since the students selected, consumed, and enjoyed minimally processed dried apple chips less than unprocessed apples, it is evident that they did not like the physical appearance or taste of dried apples when compared to unprocessed apples. Other factors that could have influenced the results include previous exposure to apples, fruit snacks, and applesauce. Dried apple chips are not as mainstream at grocery stores compared to the other options, therefore they might have been a new snack food to a lot of the students. Also, dried apples are relatively more expensive than the other apple snack foods, which may have influenced the amount of families who were able to purchase them for their children and expose them to the snacks on an earlier or regular basis.

It is important to note that selection correlated with consumption for all of the snack food options, regardless of processing level. It was originally hypothesized that the students would select equal amounts of all products but consume greater amounts of more-processed options. When students had the ability to choose the portion size, they selected and consumed larger quantities of food than the recommended serving size. These results support individually packaged snack foods and smaller servings for adolescents in an effort to avoid overeating. This is particularly true for ultra-processed products that are higher in calories, fat, sugar, and sodium while being lower in fiber, protein, vitamins, and nutrients.

### 4.1. Recommendations for the Snack Food Environment

Based on this research, it is recommended that changes be made to improve the food environment and adolescent snack food trends in America. First, since the subjects were drawn to and selected greater quantities of ultra-processed foods, it is necessary to improve the appeal of nutritious snack food items that are less processed. In order to efficiently do so, government food policies, labeling laws, food safety standards, and food company values all need to be shifted to encourage health and sustainability. With a collaborative effort, snack food product development, marketing, advertising, packaging, and distribution can all move to support positive growth in the American food system. An example of this effort includes increasing advertising for whole foods such as vegetables, fruits, nuts, and seeds for snack foods. Also, food labels can be monitored and redesigned to highlight the artificial ingredients and provide consumers with simplified nutrition information and realistic portion sizes.

Since it was also identified that the subjects enjoyed the taste of ultra-processed snack foods compared to less-processed options, it is evident that the taste of less-processed snack foods should be enhanced. Food companies can do this by working with their research and development teams to create flavorful and equally tasty products without chemicals, preservatives, and other artificial ingredients. This can be achieved by experimenting with natural spices and flavors or cooking techniques. Food science and research goals should always prioritize consumer safety and health. Also, flashier packaging, unique labels, and naturally derived coloring could be used to excite adolescents about less-processed and more nutrient-dense snacking options.

It is also recommended to improve snacking in America by teaching citizens how to produce and create healthier snack foods on their own. This would improve access to healthy snack foods and reduce prices for families living in food deserts, which are defined as locations lacking access healthful and affordable foods [53]. If Americans were encouraged to grow their own food and create snacks on their own, they would be able to provide healthy snacks for their families. Examples include drying local meat and making spiced jerky, growing fruits for fresh, dried, mashed, or frozen fruit snacks, growing vegetables to be snacked on with hummus or baked into chips, and roasting nuts and seeds with cultural spices. Snack foods can be prepared at home and packaged using reusable containers to be brought on-the-go.

Lastly, educating Americans about nutrient density, how to evaluate snack food products based on nutrition, and the dangers of processing can help prepare them to handle unhealthy food environments. Peer pressure, uncertainty about what foods to consume, and misinformation about health claims can all lead to unhealthy snacking and, therefore, chronic illness and obesity. When consumers purchase snack foods, they should feel comfortable reading labels, understanding ingredients, and analyzing nutrition facts. For this to be accomplished, information about labels, food processing, and healthy snacking should be incorporated into school health classes. Government tools such as videos, pamphlets, or booklets should also be developed and widespread for families to understand snack foods. The desire to eat healthy snacks and sway consumer choice starts with education and having the knowledge to understand why ultra-processed snacks can be dangerous or unhealthy in a food environment is key to changing behavior. As Americans continue to demand healthier snacks, the food system will respond by creating, marketing, and selling less ultra-processed foods that support more sustainable diets.

### 4.2. Limitations

The limitations to this study included having a small sample of students and not having random selection or assignment of testing subjects. A sample of fourth-grade students from three schools is not representative of all fourth-grade children and it is difficult to draw causal inference. Another limitation is that every child had different levels of previous exposure to processed foods and different taste preferences. The food environment both at school and at home can have a large impact on adolescent food choices due to culture, peer pressure, food prices, and availability. Lastly, it is a limitation that only one whole food item (an apple) was used because certain subjects may have enjoyed apple-based products more than others. If a student did not enjoy apples, the results of the study would not have accurately portrayed their snack food preferences across the processed snack food continuum.

## 5. Conclusions

Overall, it was found that the processing levels of snack food items have the ability to influence adolescent taste preferences along with the selection and consumption quantities. More specifically, ultra-processed and processed foods have a large appeal for adolescents, potentially leading to overconsumption and unhealthy snacking decisions. Unprocessed and minimally processed food options are not chosen as frequently as processed and ultra-processed foods when all four processing options are made available to an audience of adolescent children.

## Figures and Tables

**Table 1:** Classification of the food processing levels adapted from Juul and Hemmingsson [13].

Classification	Description	Examples
Unprocessed	Undergone limited processing including chilling, slicing, grating, and packaging.	Fresh fruit and vegetables, unsalted nuts and seeds, grains, milk, and pulses.
Minimally Processed	A small amount of processing including drying, freezing, pasteurizing, gas and vacuum packing, fat reduction, and fermentation.	Frozen produce, dried beans, dried fruits, unsweetened fruit juices, pasteurized milk, coffee, and plain yogurt.
Processed Culinary Ingredient	Include ingredients that are originally taken from plants or nature and are then milled, pressed, pulverized, stabilized, or purified to be used in cooking or baking.	Vegetable oils, fats, butter, cream, sugar, sweeteners, salts, and flour.
Processed	Include the combination of culinary ingredients with whole foods to increase the taste or durability using processing and preservation techniques such as canning with oils, salting, pickling, smoking, and curing.	Fruits preserved in syrups, canned meats in brine, reconstituted meat, and cheese.
Ultra-Processed	Created using little whole food ingredients and a large number of additives, including artificial flavors, colors, sweeteners, emulsifiers, and preservatives. Processing techniques include baking, frying, moulding, and hydrogenation. Ultra-processed products can be fortified with micronutrients and are formulated to be “ready-to-eat” versions of whole food items.	Cereals, grain bars, hot dogs, cookies, candies, chips, crackers, soft drinks, and sauces.

**Table 2:** Demographics from the three schools involved in the research study [33,34].

School	Number of Total Students Enrolled in School	Number of Classrooms and Students Participating in Study	Race (Entire school)	Gender (Entire school)	National School Lunch Program Participation (Entire school)
1	164	2 (25 and 28 students)	92.7% Caucasian	51% Male and 49% Female	16.50%
2	161	1 (28 students)	87.6% Caucasian	42% Male and 58% Female	19.3%
3	448	1 (24 students)	89.1% Caucasian	52% Male and 48% Female	37.1%

**Table 3:** Eight apple snack food items used for the research study were randomly assigned to the classrooms so that each classroom received one product from each processing level in a randomized buffet order. Foods were classified into processing levels [13].

Food Product	Processing Level	Processing Level Criteria
Gala Apple Slices Red Delicious Apple Slices	Unprocessed	Whole food Sliced and washed
Bare Brand Dried Apple Chips UNFI Dried Apples	Minimally Processed	Dried or slowly baked Zero added ingredients
Musselman’s Cinnamon Applesauce Seneca Cinnamon Applesauce	Processed	Mashed Combined with processed culinary ingredients (sugar, water, cinnamon) Jarred for preservation
Welch’s Apple Medley Fruit Snacks (WAMFS) Western Family Fruit Snax (WFFS)	Ultra-processed	WAMFS: 18 ingredients such as artificial food coloring, corn syrup, lactic acid, ascorbic acid, and alpha tocopherol acetate [38] WFFS: 15 ingredients including corn syrup and artificial coloring [39] Artificial ingredients such as colors, preservatives, sweeteners, and stabilizers Fortified with micronutrients

**Table 4:** Names used for labeling snack food options on the buffet line.

Product Classification	Plain Name	Catchy Name
Unprocessed	Apples	All-star Apples
Minimally Processed	Dried Apples	Capitan Apple Crisps
Processed	Cinnamon Applesauce	Apple Super Sauce
Ultra-Processed	Fruit Snacks	Fruity Tooty Fruit Snacks

**Table 5:** The Tried It, Liked It, Loved It Survey.

Snack Food	Tried It	Liked It	Loved It	Did Not Try ^2^
Example entry ^1^		X		
Raw apple slices				
Dried Apples				
Cinnamon Applesauce				
Fruit Snacks				

^1^ Students were provided with the chart above and were asked to place an X in the box that explained their satisfaction with each snack food. ^2^ The categories “Tried It,” “Liked It,” “Loved It,” “Did Not Try” were pilot tested in a previous study [49] and directions were thoroughly explained to the students prior to the study. The students understood that “tried it” would be the correct answer if they did not like the food.

**Table 6:** Calculations made to compare the selection and consumption. All weight measurements were in grams and calculated for each student and food product.

Measurement	Calculation
Food Selection	(Weight of snack-filled bowl on scale) − (Scale weight after food selection is made per student)
Food Consumption	(Snack food selection weight) − (Food waste weight)
Percent Consumed	(Weight consumed)/(Weight selected)
Percent of Serving Consumed	(Weight consumed)/(Serving size weight)
Percent of Serving Selected	(Weight selected)/(Serving size weight)

**Table 7:** Results from the selection and consumption data originally recorded in grams and calculated into a percentage of a serving as specified on the product packaging.

Snack Food Item	Processing Category	Results	Serving Size
Apple	Unprocessed	• Mean of 70.5% of a serving selected • Mean of 53% of a serving consumed	Both: 4 slices or ½ an apple or 84 grams
Dried Apple	Minimally Processed	• Mean of 56.3% of a serving selected • Mean of 40.1% of a serving consumed	Bare: ½ cup or 18 grams UNFI: ½ cup or 30 grams
Apple Sauce	Processed	• Means of 87.6% of a serving selected • Mean of 72.2% of a serving consumed	Both: ½ cup or 118 grams
Fruit Snacks	Ultra-Processed	• Mean of 202.1% of a serving selected • Mean of 186.7% of a serving consumed	Western Family: one bag: 27 grams Welch’s: one bag: 24 grams

**Table 8:** Results from the “Tried It, Liked It, Loved It” Survey taken by the students to display preferences based on the snack food item and processing category.

Snack Food Item	Processing Category	Results
Apple	Unprocessed	• 6.2% did not try • 10.3% tried it • 27.8% liked it • 55.7% loved it
Dried Apple	Minimally Processed	• 9.2% did not try • 26.8% tried it • 26.8% liked it • 37.1% loved it
Apple Sauce	Processed	• 5.2% did not try • 11.3% tried it • 13.4% liked it • 70.1% loved it
Fruit Snacks	Ultra-Processed	• 3.1% did not try • 2.1% tried it • 14.4% liked it • 80.4% loved it
